# Effect of sulfonamide derivatives of *phenylglycine* on scopolamine‐induced amnesia in rats

**DOI:** 10.1002/ibra.12092

**Published:** 2023-02-14

**Authors:** Ankit Ganeshpurkar, Ravi Singh, Pratigya Tripathi, Qadir Alam, Sairam Krishnamurthy, Ashok Kumar, Sushil K. Singh

**Affiliations:** ^1^ Department of Pharmaceutical Engineering and Technology, Pharmaceutical Chemistry Research Laboratory I Indian Institute of Technology (Banaras Hindu University) Varanasi India; ^2^ Department of Pharmaceutical Engineering and Technology, Neurotherapeutics Laboratory Indian Institute of Technology (Banaras Hindu University) Varanasi Uttar Pradesh India

**Keywords:** amnesia, butyrylcholinesterase, docking, scopolamine, sulfonamide

## Abstract

Alzheimer's disease is a neurodegenerative disease responsible for dementia and other neuropsychiatric symptoms. In the present study, compounds **30** and **33**, developed earlier in our laboratory as selective butyrylcholinesterase inhibitors, were tested against scopolamine‐induced amnesia to evaluate their pharmacodynamic effect. The efficacy of the compounds was determined by behavioral experiments using the Y‐maze and the Barnes maze and neurochemical testing. Both compounds reduced the effect of scopolamine treatment in the behavioral tasks at a dose of 20 mg/kg. The results of the neurochemical experiment indicated a reduction in cholinesterase activity in the prefrontal cortex and the hippocampus. The levels of antioxidant enzymes superoxide dismutase and catalase were restored compared to the scopolamine‐treated groups. The docking study on rat butyrylcholinesterase (BChE) indicated tight binding, with free energies of −9.66 and −10.23 kcal/mol for compounds **30** and **33**, respectively. The two aromatic amide derivatives of *2‐phenyl‐2‐(phenylsulfonamido) acetic acid* produced stable complexes with rat BChE in the molecular dynamics investigation.

## Graphical abstract

In scopolamine‐induced amnestic rats, the sulfonamide‐based compounds, butyrylcholinesterase (BChE) inhibitors, led to improved alteration and novel arm entries in the Y maze, and improved latency time and reduced errors in the Barnes maze task. Neurochemical analysis showed inhibition of cholinesterase activity in the rat brain. The in silico analysis showed stable BChE inhibition with the use of the test compounds.

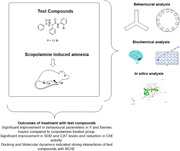

## 1 INTRODUCTION

Alzheimer's disease (AD), the most common form of dementia, results from progressive irreversible neurodegeneration in the brain.^1^ The disease typically affects short‐ and long‐term memory along with a disturbance in the circadian rhythm, agitation, apathy, that is, decreased movement and motivation, depression, confusion, delirium, speech loss, and gradual loss of body functions.^2^ However, mild cognitive impairment is the first clinical symptom. The classical hallmarks of AD include extracellular amyloid β (Aβ) plaques and intracellular neurofibrillary tangles (NFTs) of hyperphosphorylated tau proteins, which hamper normal neuronal functioning, resulting in cognitive decline.^3^ The accumulated neuritic plaques contain one or more variants of Aβ_1–42_ generated by the dysregulation of the metabolism of the amyloid precursor protein.^4^ The brain imaging of AD patients showed a reduction in hippocampal volume and enlargement in the ventricles. Furthermore, physiological alterations in synaptic function, plasticity, and neuronal excitation/inhibition have also been reported in AD.^5^ There are various hypotheses regarding the pathology and progression of the disease, including the Aβ cascade, tau hypothesis, oxidative stress, neuroinflammation, glucose hypermetabolism, cholinergic disruption and loss, gut microbiome, and bacteria‐derived metabolites.^6^ Some therapeutic targets under clinical investigation comprise β‐secretase 1, glycogen synthase kinase 3β, monoamine oxidase B, matrix metalloproteases, N‐methyl d‐aspartate receptors, and tau kinase.^7^

The entire cortex, hippocampus, and cholinergic system have a critical function in memory, learning, and cognitive process. Davies and Maloney initially proposed the role of the cholinergic system in AD in 1976. The group observed a reduction in choline acetyltransferase (ChAT) activity in the amygdala, cortex, and hippocampus in comparison to the other regions of the brain of AD patients.^8^, ^9^ ChAT is a crucial enzyme for synthesizing acetylcholine (ACh) in the synaptic terminal of cholinergic nerve fibers. The administration of anticholinergic drugs on human subjects shows reduced cognitive function and poor activities of daily living (ADL).^10^, ^11^ The patients with bilateral hippocampal injury, induced due to herpes, surgery, simplex encephalitis, or anoxia, experience amnesia, indicating the role of the neuronal tracts of the hippocampus in memory functioning. Furthermore, the disruption in cholinergic signaling in hippocampal CA3 neurons has a deleterious effect on information processing and memory formation.^12^ The impaired cholinergic signaling in the cerebral cortex causes reduced attention and decision‐making.^13^ The administration of centrally acting cholinesterase inhibitors, namely donepezil (DNP) and galantamine, improves the ADL and complex higher‐order skills in AD patients.

Among various targets, inhibition of acetylcholinesterase (AChE) is still a major component of anti‐AD therapy to provide symptomatic relief. Furthermore, DNP, an AChE inhibitor that also binds to the peripheral anionic site, inhibits Aβ aggregation as well and slow down the progression of the disease. The cholinesterases, AChE, and butyrylcholinesterase (BChE) are responsible for the synaptic catabolism of ACh and the termination of its action. In a healthy brain, AChE predominates, and BChE plays a minor role in regulating Ach.^14^ BChE is present in the amygdala, hippocampus, and neocortex, where it is usually linked to glial, vascular, and neuronal cells. A genetic knockout (KO) investigation on mice reported that the cholinergic activity of the brain remained normal after AChE KO, implying that BChE was responsible for the activity.^15^ Another study observed lower levels of amyloid fibrillar formations in the cerebral cortex in 5XFAD/BChE‐KO mice.^16^ The BChE to AChE ratio increases from 1:5 to 11:1 due to cholinergic neuronal loss in AD patients.^17^, ^18^ Ethopropazine, a selective BChE inhibitor, produced cognitive improvement in a clinical study.^19^

**Figure 1:**
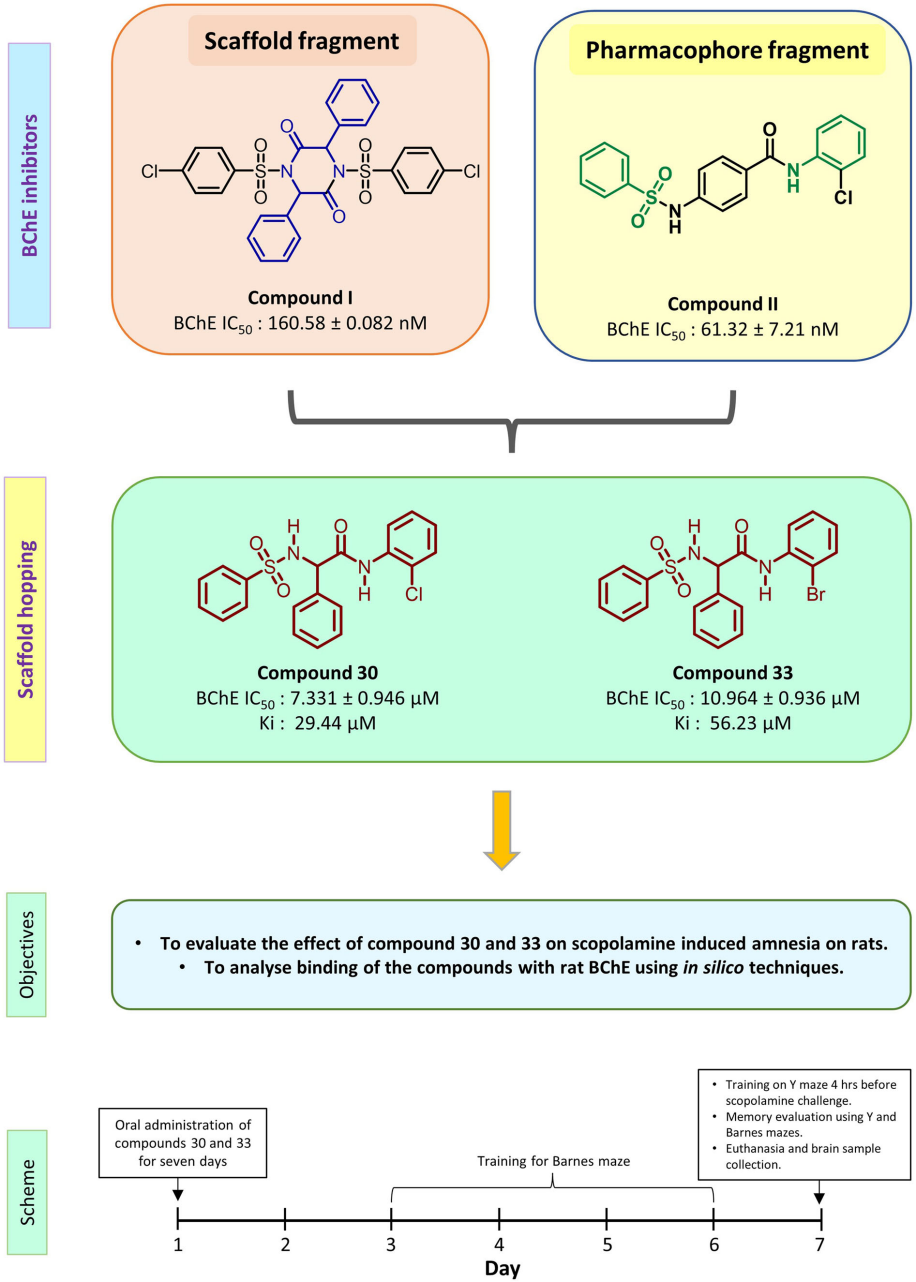
Schematic representation of the rationale and plan of the study. BChE, butyrylcholinesterase; IC_50_, half maximal inhibitory concentration. [Color figure can be viewed at wileyonlinelibrary.com]

In our previous study, we identified sulfonamide‐based compounds **I** and **II** as cholinesterase inhibitors and these were evaluated for their anti‐AD activity. Compound **I** is a sulfonamide‐based piperazinedione derivative with both AChE and BChE inhibition properties. In scopolamine‐induced amnesia in mice, the compound also induced a significant improvement in spontaneous alteration.^20^ Compound **II** was a selective BChE inhibitor.^21^ We designed sulfonamide compounds based on our previous work, and these compounds were synthesized and evaluated. Compounds **30** and **33** showed significant BChE inhibition with good blood–brain permeability^22^ (Figure 1). In the present study, the two compounds were evaluated for their effect on memory and learning in scopolamine‐induced amnesia in rats.

## 2 MATERIALS AND METHODS

### 2.1 Materials

Sodium carboxy methyl cellulose (SCMC), scopolamine hydrobromide (SCO), and DNP were procured from Sigma‐Aldrich. All the other chemicals used in the study were also of analytical grade.

### 2.2 Experimental animals

Male Wistar rats weighing 150–200 g were selected for the study. Six rats were kept in each polyacrylic cage with a 12‐h light/dark cycle at a regulated temperature (25 ± 2°C) and humidity (50 ± 10%). The animals were given free access to food and water and were acclimatized for 1 week before the experiment. Food was withheld 1 hour before the behavioral trial.

### 2.3 Experimental designs

#### 2.3.1 Drugs and treatments

The animals were divided into nine groups, each including six animals. The following treatments were included in the study: (I) control, (II) SCO (5 mg/kg), (III) SCO + DNP (5 mg/kg), (IV) SCO + compound **30** (5 mg/kg), (V) SCO + compound **30** (10 mg/kg), (VI) SCO + compound **30** (20 mg/kg), (VII) SCO + compound **33** (5 mg/kg), (VIII) SCO + compound **33** (10 mg/kg), and (IX) SCO + compound **33** (20 mg/kg). DNP and SCO were freshly dissolved in distilled water and investigational compounds were suspended in 0.5% SCMC just before the dosing. SCO was administered through intraperitoneal injection (ip), while the other compounds were administered through the oral route using oral gavage. The compounds were administered for 7 days, while SCO was administered only on the seventh day to induce amnesia. Behavioral tests were carried out half an hour after the administration of SCO.^23^

### 2.4 The lethal dose of 50% determination

The Organization for Economic Cooperation and Development (OECD) guideline 423—acute toxicity class approach was used to determine the lethal dose of 50% (LD_50_) of compounds **30** and **33**. Three female Wistar rats were used to test the compounds at 500 and 2000 mg/kg of body weight. The animals were administered doses and monitored for 72 h. The LD_50_ of the compounds was determined as per the guidelines.^24^

### 2.5 Y‐maze test

The Y‐maze is a three‐arm wooden contraption (A, B, and C) separated by a 120° angle that is used to assess the intermediate working memory and spatial memory. On the seventh (final) day of treatment, the effects of test compounds **30**, **33**, and DNP were examined. Initially, a training session was conducted in which one of the arms of the Y‐maze was closed with a wooden partition and the animal was allowed to enter the maze with the arm's head facing the center. The animal was allowed to explore the maze for 15 min during the training session. After 4 h of training and half an hour after the SCO injection, the test session was conducted. The novel arm entries and the spontaneous alteration in the three‐consecutive arms (ABC, BCA, CAB, not ABA) were considered to be indicative of memory improvements.^25^, ^26^ The maze was cleaned with 70% of ethanol after each session to eliminate any olfactory clues. The % spontaneous alteration was calculated as follows:

$$\%\;\text{Spontaneous}\;\text{alteration}=\frac{\text{Number}\;\text{of}\;\text{alteration}}{\left(\text{total}\;\text{arm}\;\text{entries}‐2\right)}\times 100.$$

### 2.6 Barnes maze

The Barnes maze is made up of 20 holes with a diameter of 10 cm, evenly spaced around the rim of a 122‐cm round gray wooden circular platform, which is raised 100 cm above the ground. The platform is lit with white light with a luminous intensity above 600 lux and a sound level of more than 80 dB.

### 2.7 Habituation

The phase involves acclimating the rats to the maze's platform and the escape box to reduce anxiety. It was performed a day before the acquisition phase. The animal was habituated for 3 min in the presence of light and without noise.

### 2.8 Acquisition phase

The acquisition phase lasted for 5 days, with one session per day. Each session included two 180‐s trials separated by 15 min. The training began with the animal being placed in the center of the platform, which was surrounded by a black box. The light and sound sources were turned on, and the animal was released after 10 s. The animal was allowed to explore the maze and use visual clues to find the location of the escape box. As soon as the rodent entered the escape box, the hole was covered and the light and sound stimuli were turned off. The animal was allowed to stay in the box for 30 s and then returned back to the cage. If the animal could not locate the escape box within 180 s, it was gently led to the box and was allowed 30 s to examine it. Olfactory stimuli were cleaned with 70% ethanol. Primary latency, the time required for a rat to reach the escape box, and primary errors, that is, the number of holes visited before reaching the escape hole was recorded.

### 2.9 Probe trial

The probe trial was conducted on the seventh day of treatment and 30 min after the ip administration of the SCO. The probe trial was conducted in a similar manner, with the escape holes blocked. The rat was given 90 s to explore the maze, and primary latency and errors were recorded.^27^, ^28^

### 2.10 Neurochemical analysis

The animals were euthanized and the brains were extracted after the experiments. A neurochemical study was performed on the hippocampus and the cortex. The tissue was homogenized in 10 mM phosphate‐buffered saline (PBS; pH 7.4) and centrifuged for 15 min at 4°C and 15,000 rpm; the supernatant was obtained and used for further investigation.

The cholinesterase activity was determined using the Ellman technique with acetylthiocholine iodide (ATCI) and butyrylthiocholine iodide (BTCI) as substrates. After diluting 10 µL of the supernatant with 100 µL of PBS, 50 µL of a freshly prepared substrate solution (5 mM) was added and incubated for 5 min. Furthermore, a 1.5 mM 5,5′‐dithiobis(2‐nitrobenzoic acid) solution was added, and the absorbance was measured at 415 nm, against a blank, on a Synergy HTX multimode reader (BioTek).

Catalase (CAT) is an enzyme that converts toxic H_2_O_2_ gas into water and oxygen. The CAT activity was determined by diluting 10 µL of the supernatant with 150 µL of PBS in a tissue homogenate. The reaction mixture was incubated with 250 µL of H_2_O_2_ (160 mM) for 1 min at 37°C and was heated for 15 min after adding 1.5 mL of dichromate/acetic acid stopping solution (5% K_2_Cr_2_O_7_/glacial acetic acid; 1:3 vol/vol). A green color developed from oxidation of dichromate to chromic (III) sulfate, and was compared with the control containing all the components except the enzyme. Absorbance was recorded at 570 nm on a Synergy HTX multimode reader (BioTek) against a blank.^20^

Markland's method, based on the autoxidation of pyrogallol, was used to quantify superoxide dismutase (SOD). Initially, 200 µL of 0.1 M Tris‐HCl with 1 mM ethylenediaminetetraacetic acid at pH 8.2 was added to 10 µL of tissue homogenate. This was followed by the addition of 50 µL of a 4.5 mM pyrogallol solution prepared in 1 µM HCl. The absorbance was measured after 1 min at 325 nm against a blank on a Synergy HTX multimode reader (BioTek) system. The enzyme activity was measured by using a control sample without the tissue supernatant. The experiments were carried out in triplicate, with enzyme activities being normalized against the control group.

### 2.11 Biochemical analysis

Alanine aminotransferase (serum glutamic‐pyruvic transaminase, SGPT), aspartate aminotransferase (serum glutamic‐oxaloacetic transaminase, SGOT), urea, and creatinine were determined in the serum of animals treated with compounds **30** and **33** and the control using a commercially available kit, obtained from the Tara Clinical System.

### 2.12 Statistical analysis

The results were described as mean ± standard error mean. One‐way analysis of variance was used for multiple comparisons between different groups using the Tukey test. GraphPad Prism 5 was used for data analysis. *p* < 0.05 was considered statistically significant.

### 2.13 Homology modeling

The rat BChE protein model was constructed using SWISS‐MODEL, a web server accessible via ExPASy (https://swissmodel.expasy.org/).^29^ The template search was carried out using the classical model by providing accession code—Q9JKC1 for rat BChE.^30^ The templates obtained were used to generate BChE models. Global model quality estimation (GMQE), qualitative model energy analysis (QMEAN), and the Ramachandran plot were used to measure the quality.^31^, ^32^, ^33^

### 2.14 Molecular docking

The quality of the homology model was improved by using the DOCKPREP tool of Chimera‐1.4, which fills in missing side chains and adds hydrogens and charges to the models.^34^ The PDB2PQR server (http://nbcr-222.ucsd.edu/pdb2pqr 2.1.1) was used to assign the appropriate protonation state to the amino acid residues at pH 7.4.^35^, ^36^ PDB was converted to PDBQT format using AutodcokTools‐1.5.6. The ligand structures in the SMILES format were translated into the Tripos MOL2 format using *rdkit*.^37^ They were converted into the PDBQT format using Autodock Tools‐1.5.6 after energy minimization using the MMFF94s force field. Grid maps were generated for several types of atoms present in the ligands (A, C, HD, NA, S, N, OA, Cl, Br, and I) using Autogrid‐4.2.6. A grid box 84 × 66 × 72 in size was used, and the grid center was placed at 51.05, 28.382, and 54.297, representing *X*, *Y*, and *Z* coordinates, respectively, with a grid point spacing of 0.375 Ǻ.^38^ The Lamarckian genetic algorithm was used along with the Solis–Water local search to identify various conformations of a ligand, which were then scored using the native scoring function of Autodock‐4.2.6. A python script, vstools v0.16, was used to process the docking findings, and Discovery Studio Visualizer 2020 was used to perform postdocking analysis and visualization.^39^

### 2.15 Molecular dynamics

The ligand parameters were generated from the docking pose of compounds **30**, **33**, and DNP through the *antechamber* module of AMBER 20. It used the general AMBER force field (GAFF2) and the Austin model with bond and charge correction (AM‐BCC1) for assigning atomic partial charges. The *tleap* module of AMBER 20 was used to produce the corresponding coordinate and topology data for protein and protein–ligand complexes. The resulting complexes were hydrated in a cubic box containing TIP3P water molecules, with a cutoff distance of 12 Å between any atom in the protein and the periodic boundary's edge. The system was then neutralized by adding Na+ and Cl− ions. It was subjected to energy minimization, heating, density equilibration, and equilibration under periodic boundary conditions.^40^, ^41^, ^42^ The final molecular dynamics (MD) were carried out at 310.15 K as an NPT ensemble for 50 ns.^43^, ^44^, ^45^ The post‐MD processing was carried out using *cpptraj*.^46^

## 3 RESULTS

### 3.1 LD_50_ determination

The OECD guideline 423—acute toxicity class approach was used. The LD_50_ values for compounds **30** and **33** were found to be 300 and 1000 mg/kg, respectively (Supporting Information: Tables T1–T12).

### 3.2 Y‐maze

**Figure 2:**
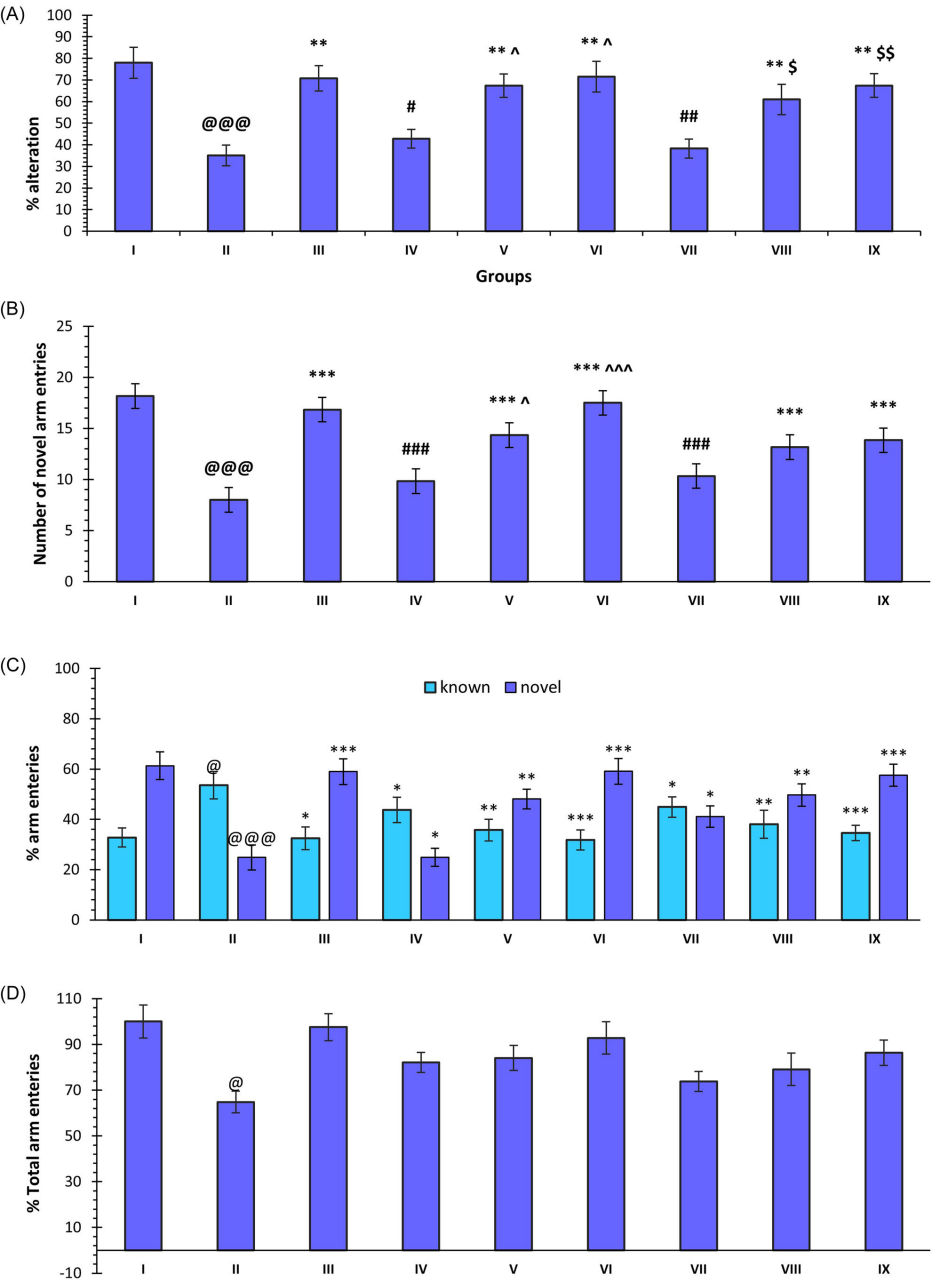
Effect of compounds **30, 33**, and DNP on (A) scopolamine‐induced impairment of % spontaneous alteration, (B) novel arm entries, (C) % arm entries, and (D) % total arm entries. Data are described as mean ± SEM (*N* = 6), one‐way ANOVA, followed by the Tukey test. ^@^ *p* < 0.05 compared to the control, p@@@ < 0.001 compared to the control, *p < 0.05 compared to SCO, **p < 0.01 compared to SCO, ***p < 0.001 compared to SCO, ^#^ *p* < 0.05 compared to DNP, ^##^ *p* < 0.01 compared to DNP, ^###^ *p* < 0.001 compared to DNP, ^^^ *p* < 0.05 compared to compound **30** (5 mg/kg), ^^^^^ *p* < 0.001 compared to compound **30** (5 mg^/^kg), ^$^ *p* < 0.05 compared to compound **33** (5 mg/kg), ^$$^ *p* < 0.01 compared to compound **33** (5 mg/kg). ANOVA, analysis of variance; DNP, donepezil; SCO, scopolamine hydrobromide. [Color figure can be viewed at wileyonlinelibrary.com]

The treatment with SCO showed a significant decrease in spontaneous alteration in the rat as compared to the control (*p* < 0.001, group: II vs. I). The treatment with DNP resulted in a significant improvement in % spontaneous alteration by reducing the effect of the SCO treatment (*p* < 0.01, group: III vs. II). In the case of compound **30**, there was a significant increase in spontaneous alteration, except at the dose of 5 mg/kg. The spontaneous alterations at doses of 10 and 20 mg/kg were significantly higher than that of SCO (*p* < 0.01, groups: V vs. II and VI vs. II), whereas a dose of 5 mg/kg of compound **30** produced less alteration (*p* < 0.05, groups: V vs. IV and VI vs. IV). Compound **33** also showed a similar trend. It was observed that the doses of 10 and 20 mg/kg produced significantly higher spontaneous alterations than SCO treatment (*p* < 0.01, groups: VIII vs. II and IX vs. II). The improvement in alteration was less at the dose of 5 mg/kg (*p* < 0.05, group: VIII vs. VII and *p* < 0.01, group: IX vs. VII) (Figure 2A).

SCO treatment lead to a significant reduction in the novel arm entries as compared to the control group (*p* < 0.001, group: II vs. I). DNP produced a significant improvement in novel arm entries in comparison to SCO treatment (*p* < 0.001, group: III vs. II). The treatment with compound **30**, at doses of 10 and 20 mg/kg, resulted in significant improvement in the novel arm entries as compared with SCO (*p* < 0.001, groups: V vs. II and V vs. II), but the effect was reduced at a dose of 5 mg/kg (*p* < 0.05, groups: V vs. IV and VI vs. IV). Similarly, compound **33** also induced a significant improvement in novel arm entries at doses of 10 and 20 mg/kg as compared to SCO (*p* < 0.001, groups: VIII vs. II and IX vs. II) (Figure 2B).

The SCO treatment group showed a significant increase in the % known arm entries in comparison to the control (*p* < 0.05, group: II vs. I). In the case of compounds **30** and **33**, an increase was observed at a dose of 5 mg/kg. On the other hand, there was no significant change in known arm entries in any of the treatment groups, as compared to the control group. The treatment with SCO led to a significant reduction in the arm entries, as compared to the control (*p* < 0.001, group: II vs. I). Further, the groups treated with compounds **30**, **33**, and DNP also showed a significant improvement in % novel arm entries as compared to SCO treatment (*p* < 0.001, group: III vs. II, VI vs. III and X vs. III; *p* < 0.01, V vs. II, VIII vs. II; and *p* < 0.05, IV vs. II, VII vs. II) (Figure 2C).

SCO treatment was found to induce a significant reduction in total arm entries, as compared to the control group (*p* < 0.05, group: II vs. I). On the other hand, none of the other treatment groups showed any significant change with respect to the control as well as SCO treatment groups (Figure 2D).

### 3.3 Barnes maze

**Figure 3:**
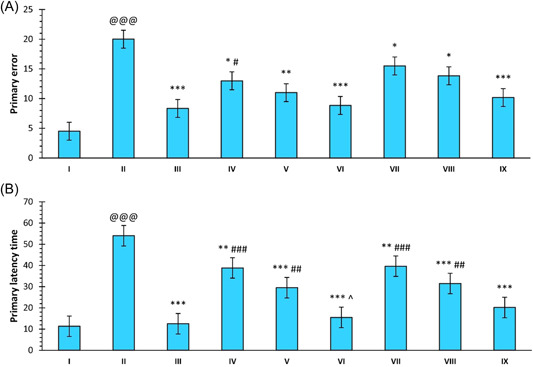
Effect of compounds **30, 33**, and DNP on (A) primary errors and (B) primary latency time. Data are described as mean ± SEM (*N* = 6), one‐way ANOVA, followed by the Tukey test. p@@@ < 0.001 compared to the control, *p < 0.05 compared to SCO, **p < 0.01 compared to SCO, ***p < 0.001 compared to SCO, ^#^ *p* < 0.05 compared to DNP, ^##^ *p* < 0.01 compared to DNP, ^###^ *p* < 0.001 compared to DNP, ^^^ *p* < 0.05 compared to compound **30** (5 mg/kg). ANOVA, analysis of variance; DNP, donepezil; SCO, scopolamine hydrobromide. [Color figure can be viewed at wileyonlinelibrary.com]

The effect of the treatment of SCO on the primary error was significantly high as compared to the control group (*p* < 0.001, group: II vs. I). The treatment with DNP caused a significantly lower primary error in the rats compared to SCO (*p* < 0.001, group III vs. II). Compound **30** was found to induce a significant reduction of primary errors as compared to SCO (*p* < 0.05, group: IV vs. II; *p* < 0.01, group: V vs. II and *p* < 0.001, group: VII vs. II). Similarly, compound **33** was also found to induce a significant reduction in primary errors at all the doses in comparison to SCO (*p* < 0.05, group: VII vs. II; *p* < 0.05, group: VIII vs. II and *p* < 0.001, group: IX vs. II). However, both compounds did not induce a significant reduction in primary errors in a dose‐dependent manner (Figure 3A).

The SCO treatment group showed a significant increase in the primary latency time when compared with the control group (*p* < 0.001, group: II vs. I). There was a significant improvement in the latency period on treatment with compound **30** at all doses as compared to SCO. The reduction in time was significant at the 20 mg/kg dose as compared to the dose of 5 mg/kg for compound **30** (*p* < 0.05, group: VI vs. IV). Similarly, compound **33** also induced a significant reduction in the primary latency time at all doses. Compounds **30** and **33** at a dose of 20 mg/kg and DNP induced a significant decrease in the primary latency time as compared to SCO (*p* < 0.001, groups: III vs. II, VI vs. II and IX vs. II), with no significant difference among each other (Figure 3B).

### 3.4 Neurochemical analysis

The total cholinesterase activity was determined for the hippocampus and prefrontal cortex (PFC) regions with two different substrates, that is, ATCI and BTCI. The total cholinesterase activity of both regions was found to be significantly elevated with ATCI as a substrate for SCO treatment in comparison to the control (*p* < 0.001, group: II vs. I for both). DNP caused a significant reduction in enzymatic activity with ATCI as a substrate as compared to SCO (*p* < 0.001, group: III vs. I). In the case of the hippocampus, all the selected doses of both compounds **30** and **33** showed no significant difference when compared with the SCO treatment. However, a significant difference was observed for all doses of both compounds with the DNP treatment. On the other hand, the treatment with 10 and 20 mg/kg doses of both compounds caused a significant reduction in the activity of enzymes as compared to the SCO treatment (*p* < 0.05, groups: V vs. II, VI vs. II, VII vs. II, and IX vs. II). While a dose of 5 mg/kg of the compounds did not show a significant difference in enzyme activity as compared to SCO, a significant difference was observed when it was compared with the DNP treatment (*p* < 0.05, groups: IV vs. III and VII vs. III).

**Figure 4:**
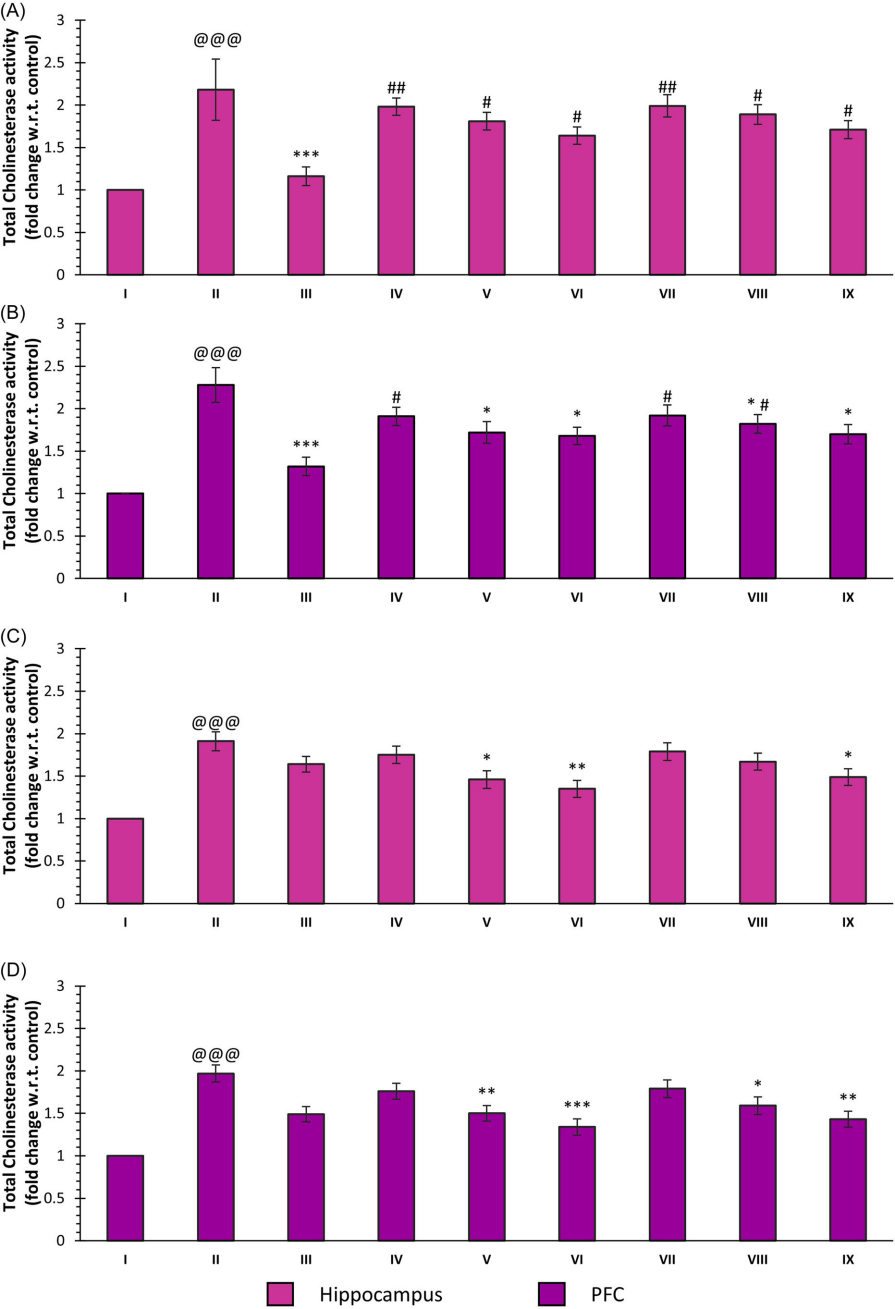
Effect of compounds **30, 33**, and DNP on total cholinesterase activity with ATCI as a substrate in (A) the hippocampus and (B) PFC and with BTIC as a substrate in (C) the hippocampus and (D) PFC. Data are described as mean ± SEM (*N* = 6), one‐way ANOVA, followed by the Tukey test. p@@@ < 0.001 compared to the control; *p < 0.05 compared to SCO, **p < 0.01 compared to SCO, ***p < 0.001 compared to SCO, ^#^ *p* < 0.05 compared to DNP, ^##^ *p* < 0.01 compared to DNP. ANOVA, analysis of variance; ATCI, acetylthiocholine iodide; BTCI, butyrylthiocholine iodide; DNP, donepezil; PFC, prefrontal cortex; SCO, scopolamine hydrobromide. [Color figure can be viewed at wileyonlinelibrary.com]

The total cholinesterase activity determined with BTCI as the substrate also indicated a significant increase in enzymatic activity in the hippocampus and PFC as compared to the control group (*p* < 0.001, group: II vs. I for both regions). In hippocampal samples, the treatment with compound **30** caused a significant reduction in cholinesterase activity with BTCI as a substrate at doses of 10 and 20 mg/kg (*p* < 0.05, groups: V vs. II, VI vs. II). Compound **33** also induced a significant reduction in the total cholinesterase activity at the 20 mg/kg dose in comparison to SCO (*p* < 0.05, group: IX vs. II). There was a significant effect on the treatment groups of 5 mg/kg for both compounds and 10 mg/kg for compound **33**. Further, a dose of 5 mg/kg of the two compounds did not lead to a significant difference in the enzyme activity when compared with the SCO treatment in PFC. The doses of 10 and 20 mg/kg showed a significant difference in enzymatic activity, with BTCI as a substrate, when compared with the SCO treatment in PFC (*p* < 0.01, group: V vs. II; *p* < 0.001, group: VI vs. II; *p* < 0.05, group: VIII vs. II and *p* < 0.01, group: IX vs. II) (Figure 4).

**Figure 5:**
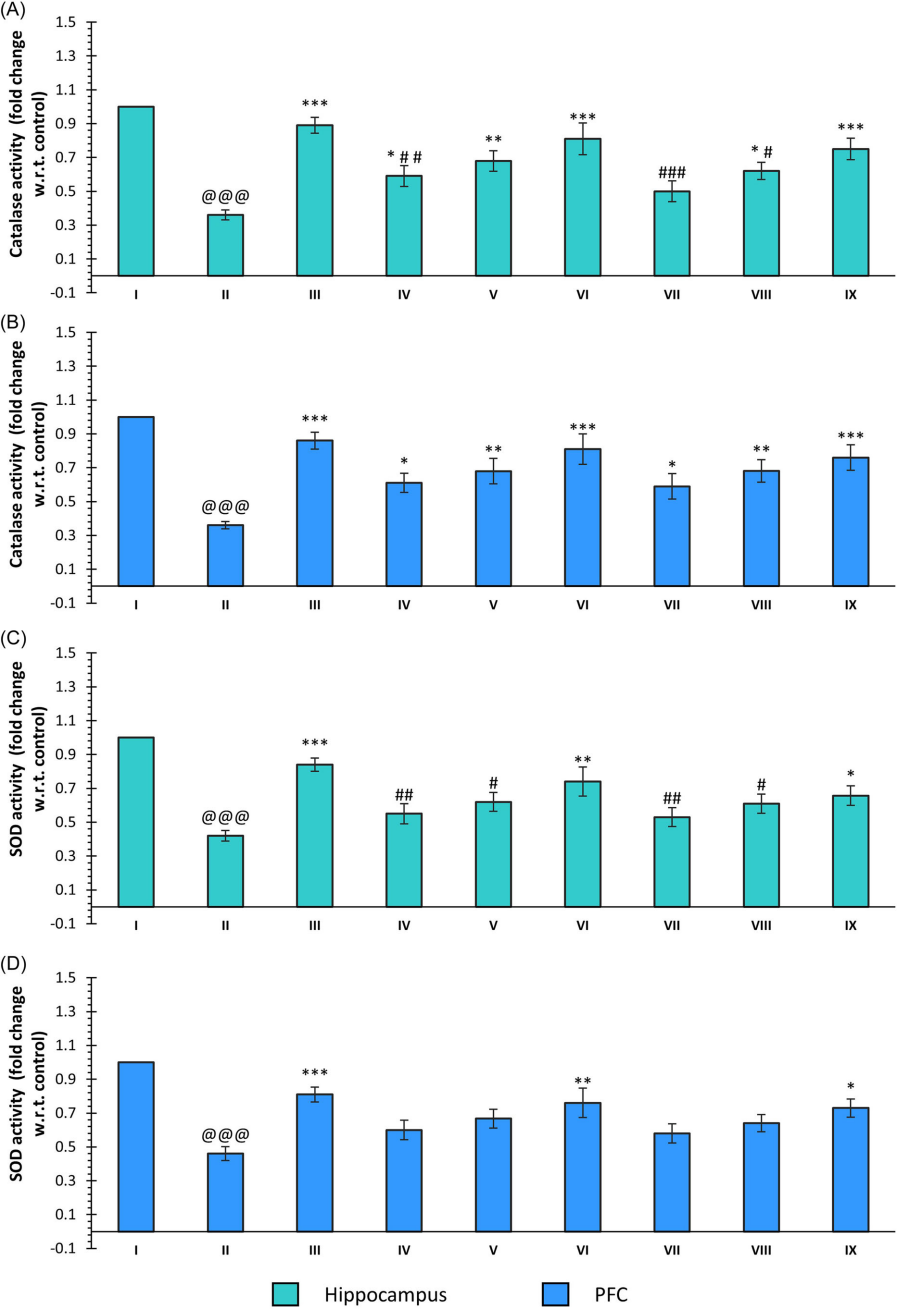
Effect of compounds **30, 33**, and DNP on CAT activity in (A) the hippocampus and (B) PFC and SOD activity in (C) the hippocampus and (D) PFC. Data are described as mean ± SEM (*N* = 6) one‐way ANOVA, followed by the Tukey test. p@@@ < 0.001 compared to the control, *p < 0.05 compared to SCO, **p < 0.01 compared to SCO, ***p < 0.001 compared to SCO, ^#^ *p* < 0.05 compared to DNP, ^##^ *p* < 0.01 compared to DNP, ^###^ *p* < 0.001 compared to DNP. ANOVA, analysis of variance; DNP, donepezil; PFC, prefrontal cortex; SCO, scopolamine hydrobromide; SOD, superoxide dismutase. [Color figure can be viewed at wileyonlinelibrary.com]

CAT activity was significantly reduced in the hippocampus and PFC regions of the SCO‐treated group when compared to the control group (*p* < 0.001, group: II vs. I). The treatment with DNP induced significantly higher activity of the CAT in both regions as compared to the SCO treatment (*p* < 0.001, group: III vs. II). In the hippocampus region, it was observed that compound **30** produced a significant increase in CAT activity at all the doses, with the maximum activity observed at the 20 mg/kg dose (*p* < 0.05, group: IV vs. II; *p* < 0.01, group: V vs. II; and *p* < 0.001, group: VI vs. II). The treatment with compound **33** was found to induce a significant improvement in the levels of CAT activity at 10 and 20 mg/kg doses (*p* < 0.05, group: VIII vs. II and *p* < 0.001, group: IX vs. II). The CAT activity of the PFC region was also found to be significantly high for all the doses of compounds **30** and **33** when compared to SCO (*p* < 0.05, group: IV vs. II and VII vs. II; *p* < 0.01, groups: V vs. II and VII vs. II; and *p* < 0.001, groups: III vs. II, VI vs. II and IX vs. II) (Figure 5A,B).

The treatment of SCO also resulted in a significant decrease in SOD activity in both the hippocampus and PFC when compared to the control (*p* < 0.001, group: II vs. I). In the case of DNP, the SOD level was significantly high in both regions as compared to the SCO treatment (*p* < 0.001, group: III vs. II). The treatment of compounds **30** and **33** at a dose of 20 mg/kg led to significantly high SOD activity as compared to SCO (*p* < 0.01, group: VI vs. II and *p* < 0.05, group: IX vs. II) in the hippocampal region. The other doses did not produce any significant increase in SOD activity. In the case of PFC, it was also found that a dose of 20 mg/kg of the both compounds induced significantly higher activity of SOD as compared to the SCO treatment (*p* < 0.01, group: VI vs. II and *p* < 0.05, group: IX vs. II) (Figure 5C,D).

### 3.5 Biochemical analysis

**Figure 6:**
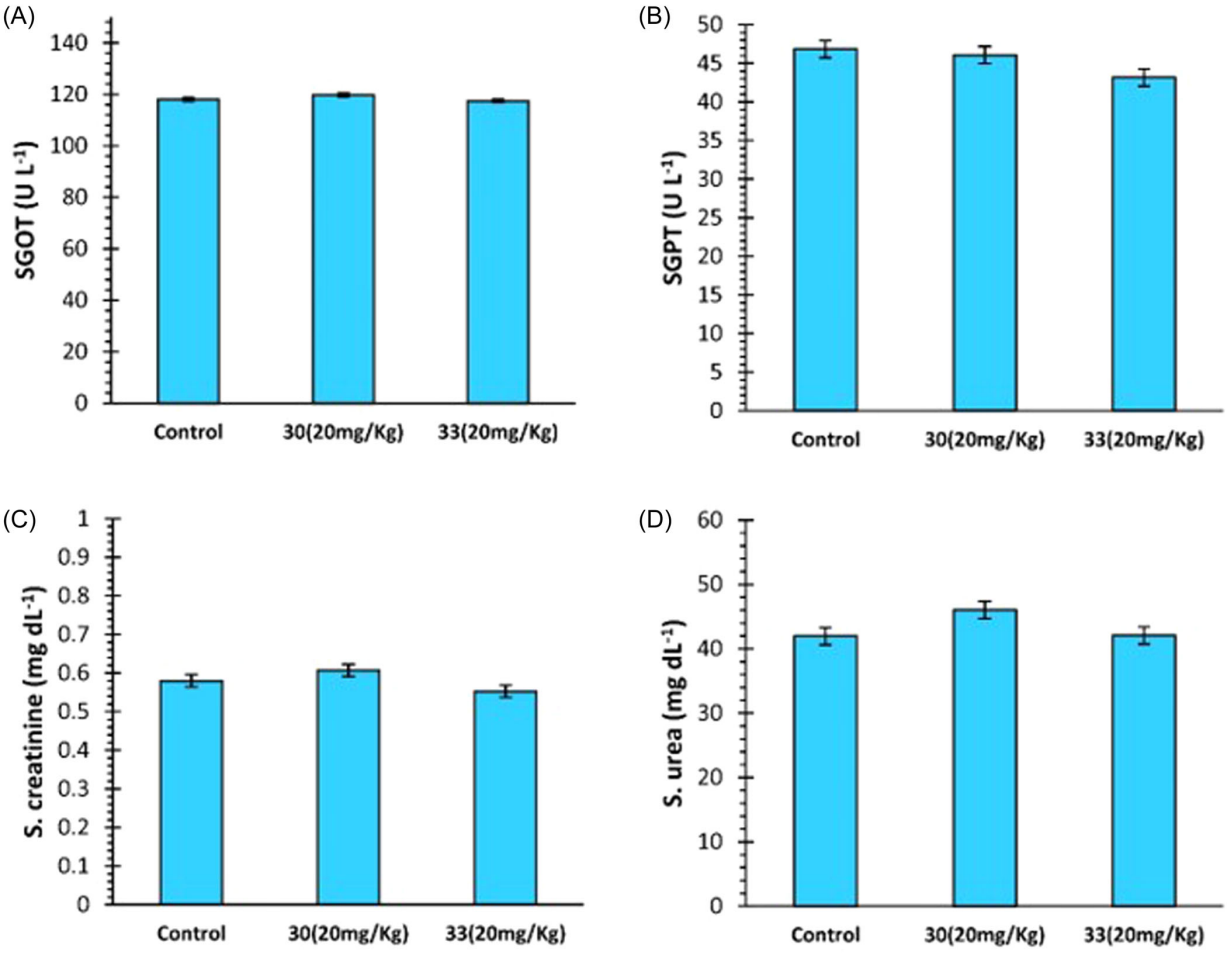
Effect of compounds **30** and **33** on (A) SGOT, (B) SGPT, (C S. creatinine, and (D) S. urea levels. Data are described as Mean ± SEM (*N* = 6) one‐way ANOVA, followed by the Tukey test. ANOVA, analysis of variance; S. creatinine, serum creatinine; S. urea, serum urea; SGOT, serum glutamic‐oxaloacetic transaminase; SGPT, serum glutamic‐pyruvic transaminase. [Color figure can be viewed at wileyonlinelibrary.com]

There was no significant difference in the levels of SGPT and SGOT enzymes between the control and the treatment groups in biochemical analysis. Similarly, no significant difference was observed in serum creatinine and urea levels among the control and treatment groups (Figure 6).

### 3.6 Homology modeling

The homology model for rat BChE was constructed using three PDB templates, shown in Table 1, obtained from the template search. The structural quality of the obtained models are also included in the table. The QMEAN score is representative of the structural properties of the protein, namely, C–β, all‐atom, solvation, and torsion potentials. The model obtained from PDB id—5LKR was the most suitable of all the models. It was further subjected to nine stages of minimization. The sixth stage of the minimization process produced the most suitable protein model with the lowest Ramachandran outlier, rotamer outlier, C–β deviation, and bad angles (Supporting Information: Figure S6).

**Table 1:** Evaluation parameters of the quality of the homology model.

Model	PDB template	Sequence similarity (%)	QMEAN	MolProbity score	Clash score	Ramachandran favored	Ramachandran outlier	Rotamer outlier	C–β deviation	Bad bonds	Bad angles
1	5LKR	79.93	−0.87	1.16	0.36	94.37	0.38	1.55	2	0	34
2	3O9M	79.93	−1.55	2.08	2.99	92.06	0.95	4.61	10	1	45
3	6I2T	79.97	−1.57	1.67	2.7	93.93	1.25	1.86	7	1	41
4	Stage 6 of energy minimization of homology model obtained from PDB—5LKR	79.93	−0.86	0.82	0	95.38	0.21	0.92	2	0	20

Abbreviations: PDB, protein data bank; QMEAN, qualitative model energy analysis.

### 3.7 Molecular docking

**Figure 7:**
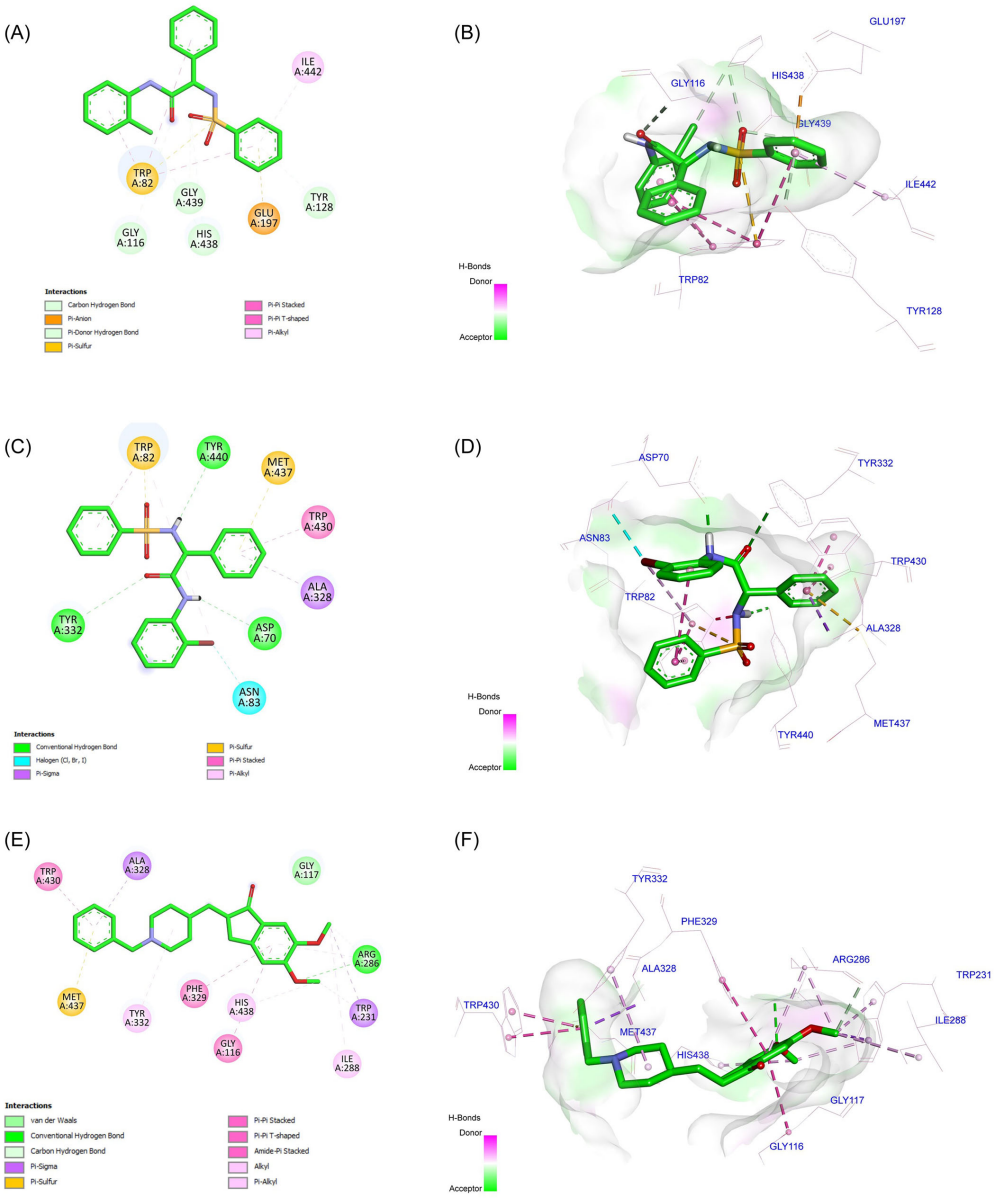
2D and 3D docking interactions of compounds (A, B) **30**, (C, D) **33**, and (E, F) DNP. 2D, two dimensional; DNP, donepezil. [Color figure can be viewed at wileyonlinelibrary.com]

The docking results indicated that the order of the binding energy was compound **32** < DNP < compound **30**. Furthermore, compound **30** showed hydrogen bonding with Gly116, Tyr128, His438, and Gly439, π–π interaction with Trp82, π–anion interactions with Trp82 and Glu197, and π–alkyl interaction with Ile442. Compound **33** interacted with Asp70, Tyr332, and Tyr440 through a hydrogen bond. It also showed π–π interactions with Trp430, π–σ interactions with Ala328, π–anion interactions with Trp82 and Met437, and halogen bonding with Asn83. DNP showed hydrogen bonding with Arg286, π–anion interaction with Met437, π–alkyl interactions with Trp231 and Ala328, π–π interactions with Gly116, Phe329, and Trp430, and π–σ interactions with Ile288, Tyr332, and His438 (Figure 7). Ligand efficiency (LE) determines binding energy per atom, and compound **33** showed the lowest LE, followed by compound **30** and DNP (Table 2).

**Table 2:** Binding energies and ligand efficiencies of various ligands against rat BChE.

Compound code	Binding energy (kcal/mol)	Ligand efficiency
**30**	−9.66	−0.36
**33**	−10.23	−0.38
DNP	−9.94	−0.36

Abbreviations: BChE, butyrylcholinesterase; DNP, donepezil.

### 3.8 MD

MD is a state‐of‐the‐art technique that is used to determine the stability of protein–ligand complexes under dynamic conditions. MD simulations of compounds **30**, **33**, and DNP were carried out under periodic boundary conditions with a water box surrounding the complex. Before the MD run, a pre‐MD phase consisting of energy minimization, temperature equilibration, and density equilibration was carried out. This was always followed by a small MD run to evaluate the behavior of the system before MD. Energy minimization was carried out to determine the proper molecular orientation in the space, which was energetically favored. It was performed using a nine‐stage process that gradually reduced the restraining weights applied on the protein's backbone to prevent an uncontrolled conformational shift (Supporting Information: Table T13). The energy minimization resulted in the change of total potential energy from −198,580, −198,480, −198,510, and −198,320 to −307,550, −307,680, −307,840, and −307,710 kcal/mol for rat BChE and rat BChE complexed with compounds **30**, **33**, and DNP, respectively. This was followed by temperature equilibration, which resulted in the increase of the temperature to 310.15 K in 5 ps for all the systems. The mean temperatures were 310.33 ± 1.07, 310.27 ± 1.16, 310.25 ± 0.96, and 310.21 ± 1.06 K for BChE and BChE complexed with compounds **30**, **33**, and DNP, respectively. Further, density equilibration, at a constant volume, was performed to obtain a unit density before the MD run. Various systems achieved a density of 1 g/cm^3^ at 50, 49, 50, and 45 ps for BChE and BChE complexed with compounds **30**, **33**, and DNP, respectively. Finally, a pre‐MD run of 1 ns at NPT was carried out on the systems. It was found that all the developed MD systems were stable, with no abrupt fluctuations in the root mean square deviation (RMSD). The mean RMSD(s) were 1.021 ± 0.191, 1.137 ± 0.191, 1.067 ± 0.135, and 1.039 ± 0.141 Å for BChE and BChE complexed with compounds **30**, **33**, and DNP, respectively (Supporting Information: Figures S1–S4).

**Figure 8:**
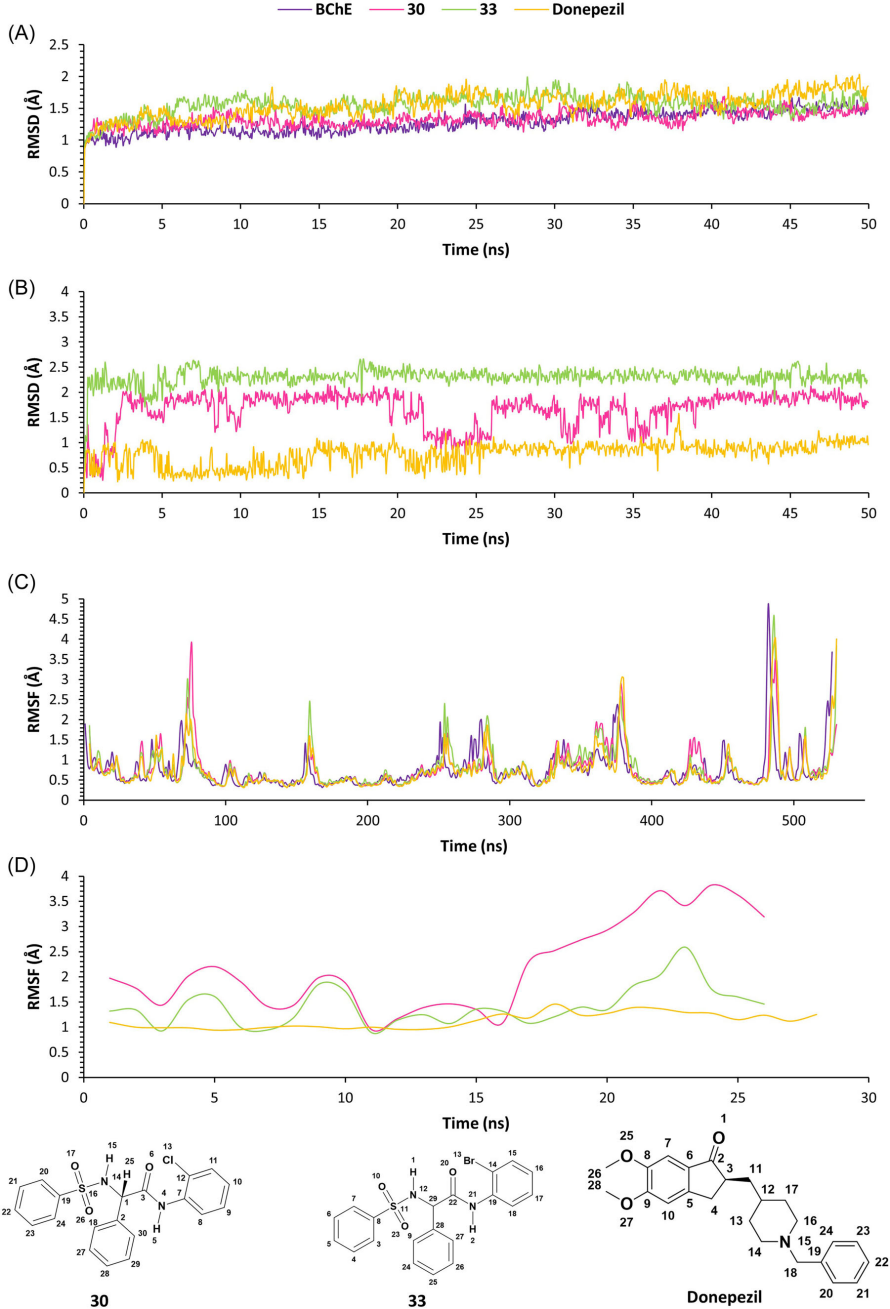
(A) RMSD of protein–ligand complexes, (B) RMSD of the ligands, (C) RMSF of protein–ligand complexes, and (D) RMSF of the ligands. RMSD, root‐mean‐square deviation; RMSF, root mean square fluctuation. [Color figure can be viewed at wileyonlinelibrary.com]

RMSD is used for the assessment of the stability of a complex during an MD run. The system is dynamic and a small structural deviation is expected during the run. RMSD of a globular protein should be between 1 and 3 Å. The mean RMSD values were 1.586 ± 0.247, 1.598 ± 0.227, 1.663 ± 0.203, and 1.528 ± 0.200 Å, for BChE and BChE complexed with compounds **30**, **33**, and DNP, respectively. The RMSD values suggested that the complexes were stable, with little variation throughout the run. RMSD of the ligands, on the other hand, revealed a distinct feature. The mean RMSD(s) were 1.483 ± 0.356, 1.331 ± 0.347, and 1.460 ± 0.379 Å, for compounds **30**, **33**, and DNP, respectively. The test compounds showed similar RMSD deviations to those of DNP. The root mean square fluctuation (RMSF) is similar to RMSD, but it shows the mean fluctuation of each protein residue or ligand atom during simulation time. Overall, the RMSF of various complexes did not differ significantly from one another. Compound **30** showed stabilization of Asp70, Trp82, Gly116, Gly117, Leu286, Leu288, and Tyr332 as compared to uninhibited BChE. Compound **33** showed stabilization of Asp70, Gly116, Gly117, and Leu286, while DNP showed stabilization of Ser198, Ala199, Leu286, Glu325, and Tyr332. Compounds **30** and **33** showed consistent fluctuations for all the atoms. However, the *N‐benzyl* ring and *methoxy* groups showed more deviation than the other atoms in DNP (Figure 8).

The accessibility of the solvent molecules, that is, water, is measured by the solvent‐accessible surface area (SASA). The mean SASA(s) were found to be 20635.446 ± 361.385, 20145.217 ± 368.284, 20350.486 ± 466.642, and 20731.917 ± 394.768 Å^2^ for BChE and BChE complexed with compounds **30**, **33**, and DNP, respectively. A slight decrease in SASA was observed with the binding of compounds **30** and **33** as compared to protein and an increase in SASA was observed in the case of the DNP complex. The mean SASA(s) were found to be 89.053 ± 24.389, 123.983 ± 24.812, and 93.038 ± 26.309 Å^2^ for compounds **30**, **33**, and DNP, respectively. The SASA for compound **30** was the lowest among all. The fluctuation in SASA was quite significant initially and indicated that the ligands were solvent‐exposed. The radius of gyration (RoG) measures protein stability, and an increase in its value indicates that the protein is unfolded. The mean RoG(s) were 22.998 ± 0.062, 23.030 ± 0.065, 22.039 ± 0.073, and 22.092 ± 0.052 Å, for BChE and BChE complexed with compounds **30**, **33**, and DNP, respectively. No significant difference was observed in RoG(s) among the various complexes. The mean RoG(s) for compounds **30**, **33**, and DNP were 3.919 ± 0.091, 4.135 ± 0.085, and 4.408 ± 0.158 Å, respectively. There was no appreciable hydrogen bonding observed in any of the ligands, indicating that ligand binding was dominated by π interactions (Supporting Information: Figure S5).

## 4 DISCUSSION

AD is one of the most common forms of dementia, caused by the development of neuritic plaques and NFTs leading to neurodegeneration. The progression of the disease results in poor ADL, culminating in a vegetative state and thus making the life of the patient and caregiver very challenging. The current treatment strategy for AD includes the use of AChE inhibitors, that is, DNP, galantamine, and so forth. They increase the synaptic concentration of ACh and compensate for the decrease in number of cholinergic neurons. Hippocampal neuronal activation in the diseased brain results in improvements in memory, learning, and cognition. SCO is a muscarinic antagonist that acts on the CNS and can cause memory loss.^47^, ^48^ The BChE‐inhibitory activity of compounds **30** and **33** were evaluated to determine their effect on memory.

The spontaneous alternation is based on the exploratory behavior of rodents in the Y‐maze. Rodents explore a new spaces and hence they would enter a recently less visited arm of the maze.^49^ The treatment with 10 and 20 mg/kg doses of both the test compounds showed significant improvement in spontaneous alteration as compared to the SCO treatment. Further, there was no significant difference in spontaneous alteration when compared to DNP. The exploratory behavior of rodents is assessed on the basis of the novel arm entries. The novel arm entries in the case of test compounds were significantly improved at 10 and 20 mg/kg doses. However, only compound **30** led to a dose‐dependent improvement in novel arm entries. Further, there was a significant improvement in the time spent in the novel arm. The total arm entries in the Y‐maze also help in the evaluation of locomotor activity, which is highly reduced in AD patients with disease progression.^50^ Treatment with test compounds and DNP induced significant improvement in the total arm entries. The Barnes maze helps in the evaluation of spatial memory and learning. The time required to identify the target box with the help of spatial–visual clues around the maze is an important criterion. Treatment with the test compounds produced improvements in the primary latency time as compared to SCO and similar to DNP. The primary errors were also low in the case of the 20 mg/kg dose for both compounds as compared to SCO.

The efficacy of BChE inhibitors was also examined through a battery of biochemical tests carried out on PFC and hippocampal tissues isolated from the animals. The selected regions are involved in memory and cognition‐associated processes. Memory, learning, and cognition are all facilitated by cholinergic pathways and the neurotransmitter ACh. The loss of cholinergic neurons and increased cholinesterase activity are distinctly responsible for the worsening of brain function in AD. The total cholinesterase activity was determined using two substrates, that is, ATCI and BTCI. Among the two, BTCI is highly selective toward BChE. There was no significant change in the total cholinesterase activity in the presence of ATCI in the hippocampal region. In contrast, a significant reduction was observed with 10 and 20 mg/kg doses in the case of both compounds. A significant reduction in the total cholinesterase activity was observed at 10 and 20 mg/kg doses of both the compounds in PFC tissues when BTCI was used as the substrate. The total cholinesterase activity in the hippocampus was significantly reduced at 10 and 20 mg/kg doses of compound **30** and at the 20 mg/kg dose of compound **33** with BTCI as a substrate. Antioxidant enzymes such as SOD and CAT are responsible for the survival of aerobic cells, which produce reactive oxidative species.^51^ SOD neutralizes superoxide radicals into hydrogen peroxides, which are further reduced by CAT into water and oxygen. SCO administration decreased CAT and SOD activities in the brain.^52^ The CAT activity of the hippocampus and PFC significantly improved at doses of 10 and 20 mg/kg of both compounds. A significant improvement in SOD enzyme activity was observed only at a dose of 20 mg/kg. Hepatic dysfunction is reflected by alterations in the SGPT and SGOT levels. Sulfonamides, salicylates, and sulfonylureas are among the compounds that cause a moderate increase in SGPT and SGOT levels.^36^ However, no significant change was detected in the enzyme levels with both the test compounds at a dose of 20 mg/kg. Similarly, there was no significant change in serum urea and creatinine levels.

## 5 CONCLUSION

It was evident from the study that uses of test compounds **30** and **33** induced improvements in learning‐ and memory‐associated processes, when compared with SCO‐induced amnesia in rats. The compounds did not induce any alteration in SGOT, SGPT, serum urea, and creatinine levels as compared to the control group. The pharmacodynamic effect of the compounds was mainly due to the inhibition of the total cholinesterase activity in the hippocampus and PFC. The compounds also provided significant protection against SCO‐induced oxidative stress, as was reflected in the enzyme activity of SOD and CAT. Therefore, the two sulfonamide derivatives of *phenylglycine* could be considered pharmacological candidates for the treatment of AD.

## AUTHOR CONTRIBUTIONS

Ankit Ganeshpurkar and Pratigya Tripathi carried out the experiment. Ankit Ganeshpurkar, Qadir Alam, and Ravi Singh interpreted the data and arranged figures. Ankit Ganeshpurkar wrote and revised the manuscript. Sairam Krishnamurthy participated in the design and review of this study. Sushil K. Singh and Ashok Kumar were involved in the overall design and supervision of the work. All authors have read and approved the final version of the manuscript.

## CONFLICT OF INTEREST STATEMENT

The authors declare no conflicts of interest.

## ETHICS STATEMENT

This study was approved by the Institutional Animal Ethics Committee of the Banaras Hindu University, Varanasi, India (Reference no: Dean/2021/IAEC/2565). All methods were performed in accordance with relevant guidelines and regulations.

## Supporting information

Supporting information.

## Data Availability

The additional data are available in the supporting information and can also be made available by the corresponding author on reasonable request.
