# Supplementary material for: Effect of sulfonamide derivatives of phenylglycine on scopolamine‐induced amnesia in rats
Source: Ibrain. 2023 Feb 14;9(1):13–31. doi: 10.1002/ibra.12092 (PMC10529173; doi:10.1002/ibra.12092)
Supplement: Supplementary file 1 — Supporting information. [file IBRA-9-13-s001.docx]

**Supporting Information**

**Effect of sulfonamide derivatives of phenylglycine on scopolamine-induced amnesia in rats**

Ankit Ganeshpurkar^1^, Ravi Singh^1^, Pratigya Tripathi^2^, Qadir Alam^2^, Sairam Krishnamurthy^2^, Ashok Kumar^1^, Sushil Kumar Singh^1^*

^1^ Pharmaceutical Chemistry Research Laboratory I, Department of Pharmaceutical Engineering & Technology, Indian Institute of Technology (Banaras Hindu University), Varanasi -221005, India.

^2^ Neurotherapeutics Laboratory, Department of Pharmaceutical Engineering & Technology, Indian Institute of Technology (Banaras Hindu University), Varanasi-221005, U.P., India

*Corresponding author.

Sushil Kumar Singh

Pharmaceutical Chemistry Research Laboratory I, Department of Pharmaceutical Engineering & Technology, Indian Institute of Technology (Banaras Hindu University), Varanasi- 221005, India.

E-mail address: sksingh.phe@iitbhu.ac.in

**LD_50_ determination of compound 30**

**Table T1.** LD_50_ determination protocol for compound **30.**

| Test substance   1. Physical nature 2. Code | Solid  30 |
| --- | --- |
| Vehicle | 0.5% CMC solution in water |
| Test animals   1. Sex 2. Number | Rat  Female  3 |
| Test conditions   1. Dose 2. Rationale for the selection of the starting dose 3. Dosing volumes 4. Time & date of dosing | 300 mg/kg  0.7 ml  11:00 am 6/02/2021 |

**Table T2.** Effect of compound **30** on the body wt. of the animals at the dose of 300 mg/kg.

| Group | Body wt. (gm) on 6/02/2021 at 11 am | Body wt. (gm) on 7/02/2021 at 11 am | Body wt. (gm) on 8/02/2021 at 11 am | Body wt. (gm) on 12/02/2021 at 11 am | Body wt. (gm) on 20/02/2021 at 11 am |
| --- | --- | --- | --- | --- | --- |
| 1 | 236 | 230 | 229 | 234 | 238 |
| 2 | 233 | 233 | 236 | 244 | 248 |
| 3 | 224 | 221 | 223 | 229 | 231 |

**Table T3**. The onset of toxicity with compound **30** in the period of 72h.

| Group | Body wt. Changes (gm) | | | | | Onset of toxicity | Reversibility | Date & time of death |
| --- | --- | --- | --- | --- | --- | --- | --- | --- |
|  | 6/2/21 | 7/2/21 | 8/2/21 | 12/2/21 | 20/2/21 |  |  |  |
| 1 | 00 | 06 | 01 | 05 | 04 | - | - | - |
| 2 | 00 | 00 | 03 | 08 | 04 | - | - | - |
| 3 | 00 | 03 | 02 | 06 | 02 | - | - | - |

**Discussion and interpretation of results:** Animals were dosed as per the OECD guideline 423 at 300 mg/kg doses. All animals are alive at 300mg/kg dose within 72 hrs and the next 14 days.

**Conclusions:** No toxicity was observed at **300mg/kg**.

**Table T4.** LD_50_ determination protocol for the **30**.

| Test substance   1. Physical nature 2. Code | Solid  **30** |
| --- | --- |
| Vehicle | 0.5% CMC solution in water |
| Test animals   1. Sex 2. Number | Rat  Female  3 |
| Test conditions   1. Dose 2. Rationale for the selection of the starting dose 3. Dosing volumes 4. Time & date of dosing | 2000 mg/kg  No death at 300mg/kg  0.7 ml  10:00 am 9/02/2021 |

**Table T5.** Effect of compound **30** on the body wt. of the animals at the dose of 2000 mg/kg.

| Group | Body wt. (gm) on  9/02/2021at 10 am | Body wt. (gm) on  10/02/2021 at 10 am | Body wt. (gm) on  11/02/2021 at 10 am |
| --- | --- | --- | --- |
| 1 | 219 | - | - |
| 2 | 226 | - | - |
| 3 | 230 | - | - |

**Table T6**. The onset of toxicity with compound **30** in the period of 72h.

| Group | Body wt. Changes (gm) | | | Onset of toxicity | Reversibility | Date & time of death |
| --- | --- | --- | --- | --- | --- | --- |
|  | 9/2/21 | 10/2/21 | 11/2/21 |  |  |  |
| 1 | 0 | 0 | 0 | 2/02/21,1:05 pm | No | 9/02/21, 7:00 pm |
| 2 | 0 | 0 | 0 | 2/02/21, 4:30 pm | No | 9/02/21, 6:15 pm |
| 3 | 0 | 0 | 0 | - | No | 10/02/21, 9:25 am |

**Discussion and interpretation of results:** Animals were dosed as per the OECD guideline 423 at 300 mg/kg and 2000 mg/kg doses. All animals died at 2000 mg/kg dose within 72 hrs.

**Conclusions:** As per OECD guideline (Annex 2b) **LD_50_ = 500 mg/kg**.

**LD_50_ determination of compound 33**

**Table T7.** LD_50_ determination protocol for the compound **33**

| Test substance   1. Physical nature 2. Code | Solid  33 |
| --- | --- |
| Vehicle | 0.5% CMC solution in water |
| Test animals   1. Sex 2. Number | Rat  Female  3 |
| Test conditions   1. Dose 2. Rationale for the selection of the starting dose 3. Dosing volumes 4. Time & date of dosing | 300 mg/kg  0.7 ml  11:00 am 9/02/2021 |

**Table T8.** Effect of compound **33** on the body wt. of the animals at the dose of 300 mg/kg.

| Group | Body wt. (gm) on 6/02/2021 at 11 am | Body wt. (gm) on 7/02/2021 at 11 am | Body wt. (gm) on 8/02/2021 at 11 am | Body wt. (gm) on 12/02/2021 at 11 am | Body wt. (gm) on 20/02/2021 at 11 am |
| --- | --- | --- | --- | --- | --- |
| 1 | 224 | 221 | 225 | 230 | 237 |
| 2 | 242 | 240 | 241 | 243 | 246 |
| 3 | 228 | 229 | 232 | 235 | 236 |

**Table T9**. The onset of toxicity with compound **33** in the period of 72h.

| Group | Body wt. Changes (gm) | | | | | Onset of toxicity | Reversibility | Date & time of death |
| --- | --- | --- | --- | --- | --- | --- | --- | --- |
|  | 6/2/21 | 7/2/21 | 8/2/21 | 12/2/21 | 20/2/21 |  |  |  |
| 1 | 00 | 03 | 04 | 5 | 7 | - | - | - |
| 2 | 00 | 02 | 01 | 2 | 3 | - | - | - |
| 3 | 00 | 01 | 03 | 3 | 1 | - | - | - |

**Discussion and interpretation of results:** Animals were dosed as per the OECD guideline 423 at 300 mg/kg doses. All animals are alive at 300 mg/kg dose within 72 hrs and next 14 days.

**Conclusions:** No toxicity was observed at **300mg/kg**.

**Table T10.** LD_50_ determination protocol for the **33**.

| Test substance   1. Physical nature 2. Code | Solid  **33** |
| --- | --- |
| Vehicle | 0.5% CMC solution in water |
| Test animals   1. Sex 2. Number | Rat  Female  3 |
| Test conditions   1. Dose 2. Rationale for the selection of the starting dose 3. Dosing volumes 4. Time & date of dosing | 2000 mg/kg  No death at 300mg/kg  0.7 ml  10:30 am 2/02/2021 |

**Table T11.** Effect of compound **33** on the body wt. of the animals at the dose of 2000 mg/kg.

| Group | Body wt. (gm) on  9/02/2021at 11 am | Body wt. (gm) on  10/02/2021 at 11 am | Body wt. (gm) on  11/02/2021 at 11 am |
| --- | --- | --- | --- |
| 1 | 237 | 236 | 238 |
| 2 | 233 | - | - |
| 3 | 232 | - | - |

**Table T12**. The onset of toxicity with compound **33** in the period of 72h.

| Group | Body wt. Changes (gm) | | | Onset of toxicity | Reversibility | Date & time of death |
| --- | --- | --- | --- | --- | --- | --- |
|  | 9/02/21 | 10/02/21 | 11/02/21 |  |  |  |
| 1 | 0 | 1 | 1 | - | - | - |
| 2 | 0 | 0 | 0 | 9/02/21, 3:15 pm | No | 9/02/21, 4:30 pm |
| 3 | 0 | 0 | 0 | 9/02/21, 3:15 pm | No | 9/02/21, 8:00 pm |

**Discussion and interpretation of results:** Animals were dosed as per the OECD guideline 423 at 300 mg/kg and 2000 mg/kg doses. Two animals died at 2000 mg/kg dose within 72 hrs.

**Conclusions:** As per OECD guideline (Annex 2b) **LD_50_ = 1000 mg/kg**.

## Table T13: Protocol for energy minimisation carried out before molecular dynamic simulation in AMBER20.

| Stage | Maximum number of steps (steepest descent) | Maximum number of steps (conjugate gradient) | Weight for the positional restraints. (in kcal/mol-Å^2^) | Restrained elements |
| --- | --- | --- | --- | --- |
| 1 | 2000 | 3000 | 500 | Protein and ligand |
| 2 | 1000 | 4000 | 500 | Protein and ligand except for their hydrogen |
| 3 | 1000 | 4000 | 500 | Backbone of protein and ligand |
| 4 | 1000 | 4000 | 50 | Protein and ligand except for their hydrogen |
| 5 | 1000 | 4000 | 50 | Backbone of protein and ligand |
| 6 | 1000 | 4000 | 5 | Protein and ligand except for their hydrogen |
| 7 | 1000 | 4000 | 5 | Backbone of protein and ligand |
| 8 | 1000 | 4000 | 0.5 | Backbone of protein and ligand |
| 9 | 1000 | 4000 | 0.001 | Backbone of protein and ligand |


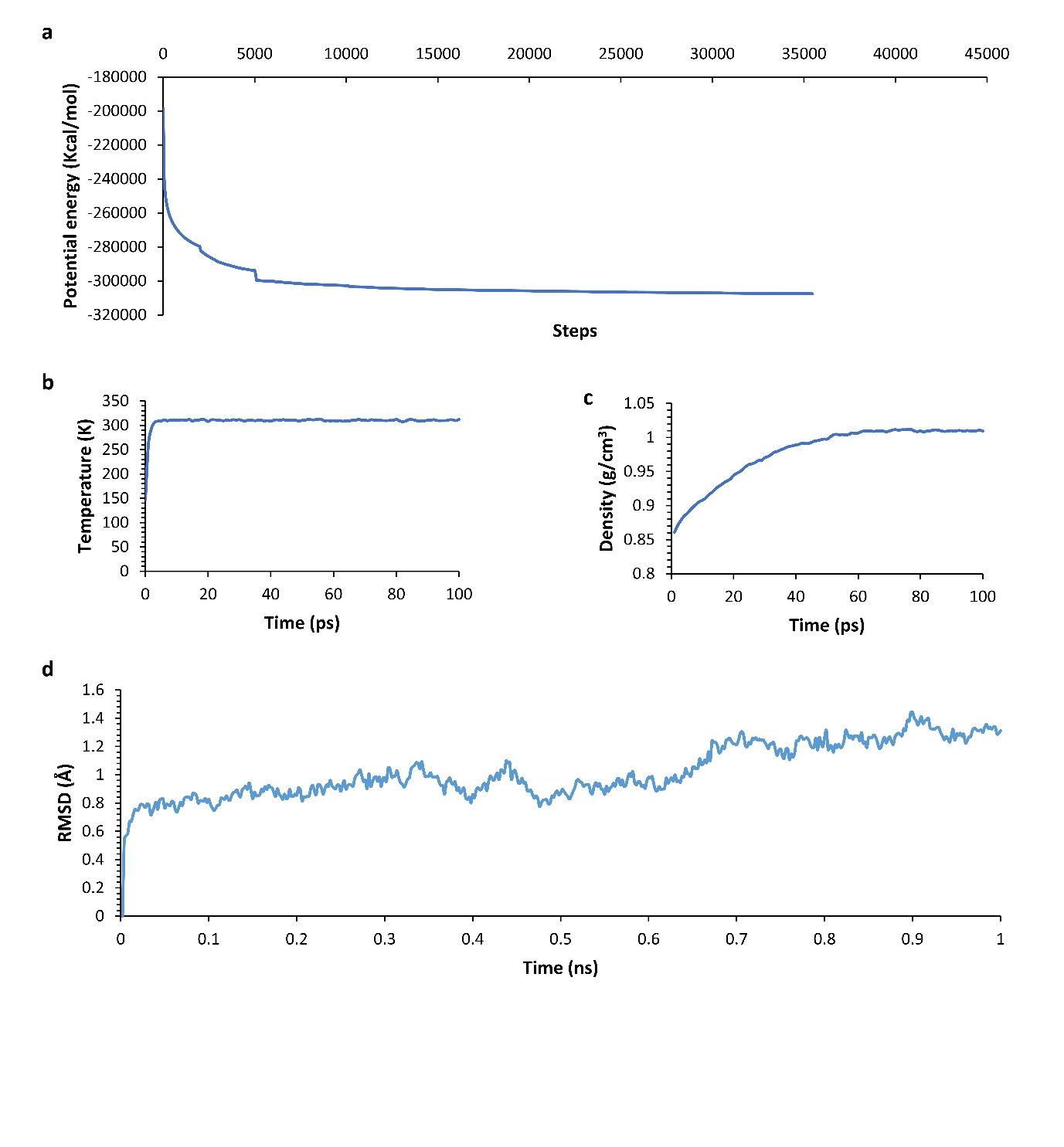


**Figure S1**: (a) Potential energy vs. Time, (b) Temperature vs. Time, (c) Density vs. Time and (d) RMSD of protein backbone of BChE.


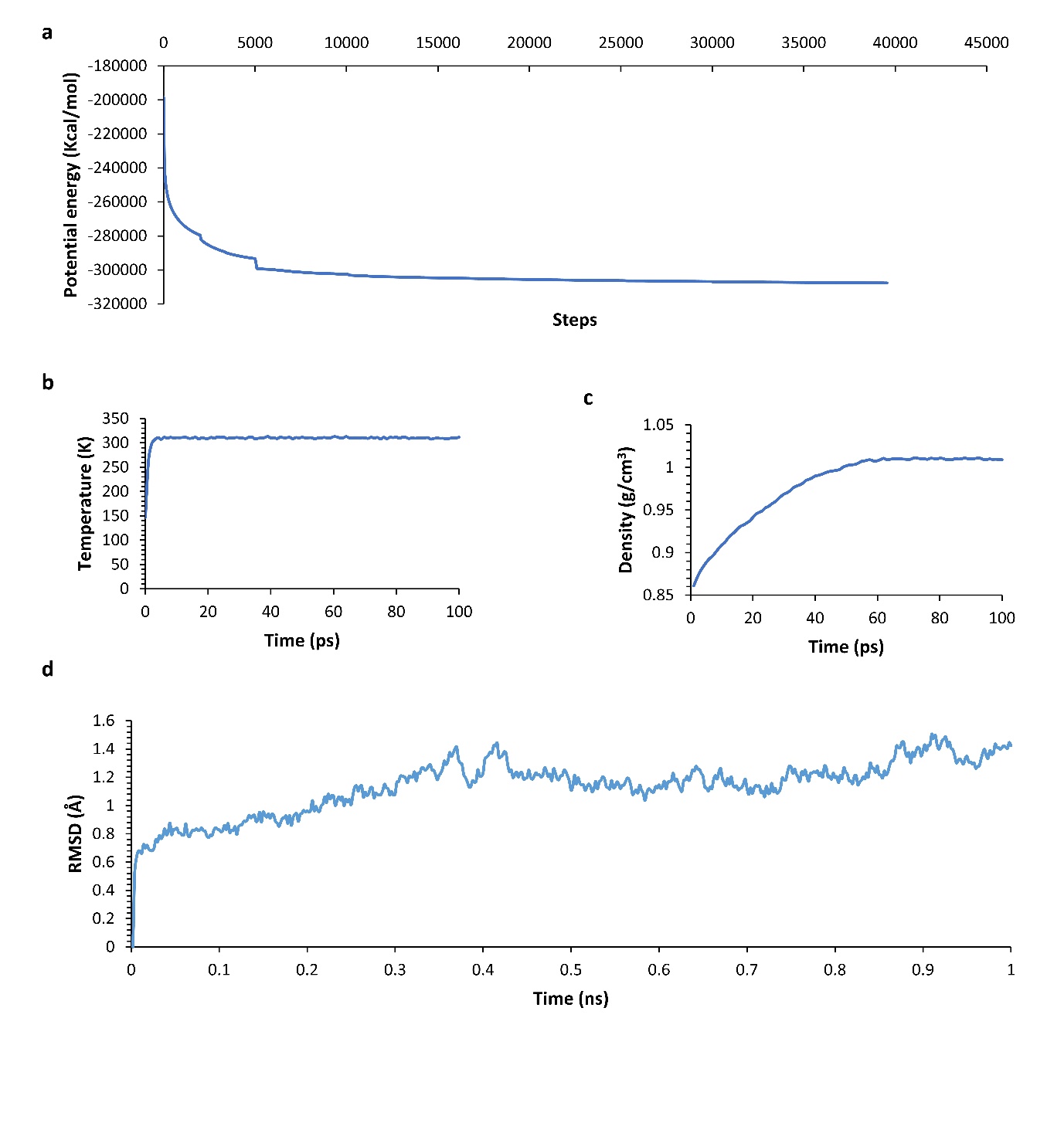


**Figure S2**: (a) Potential energy vs. Time, (b) Temperature vs. Time, (c) Density vs. Time and (d) RMSD of protein backbone of BChE-compound 30 complex.


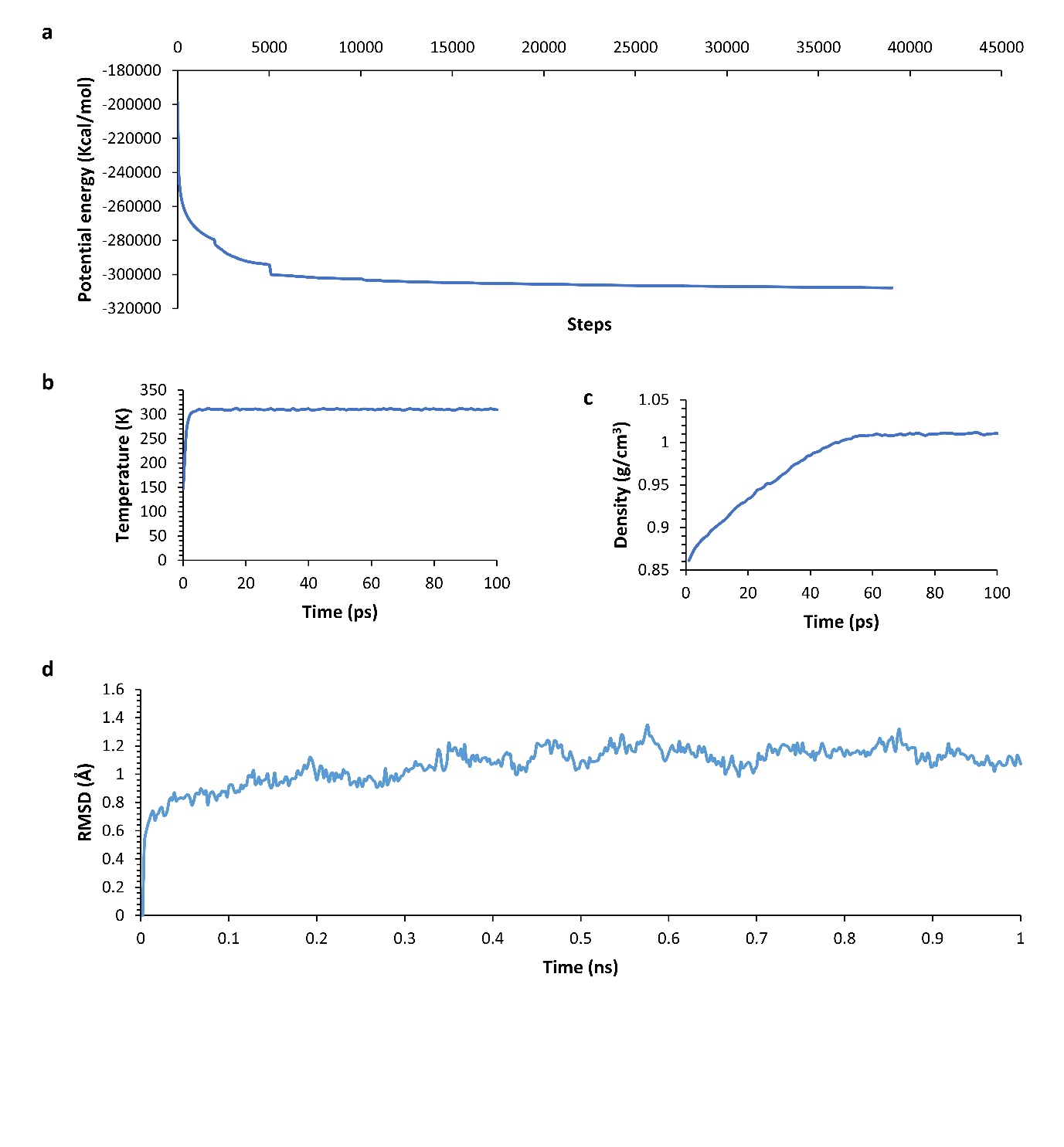


**Figure S3**: (a) Potential energy vs. Time, (b) Temperature vs. Time, (c) Density vs. Time and (d) RMSD of protein backbone of BChE-compound 33 complex.


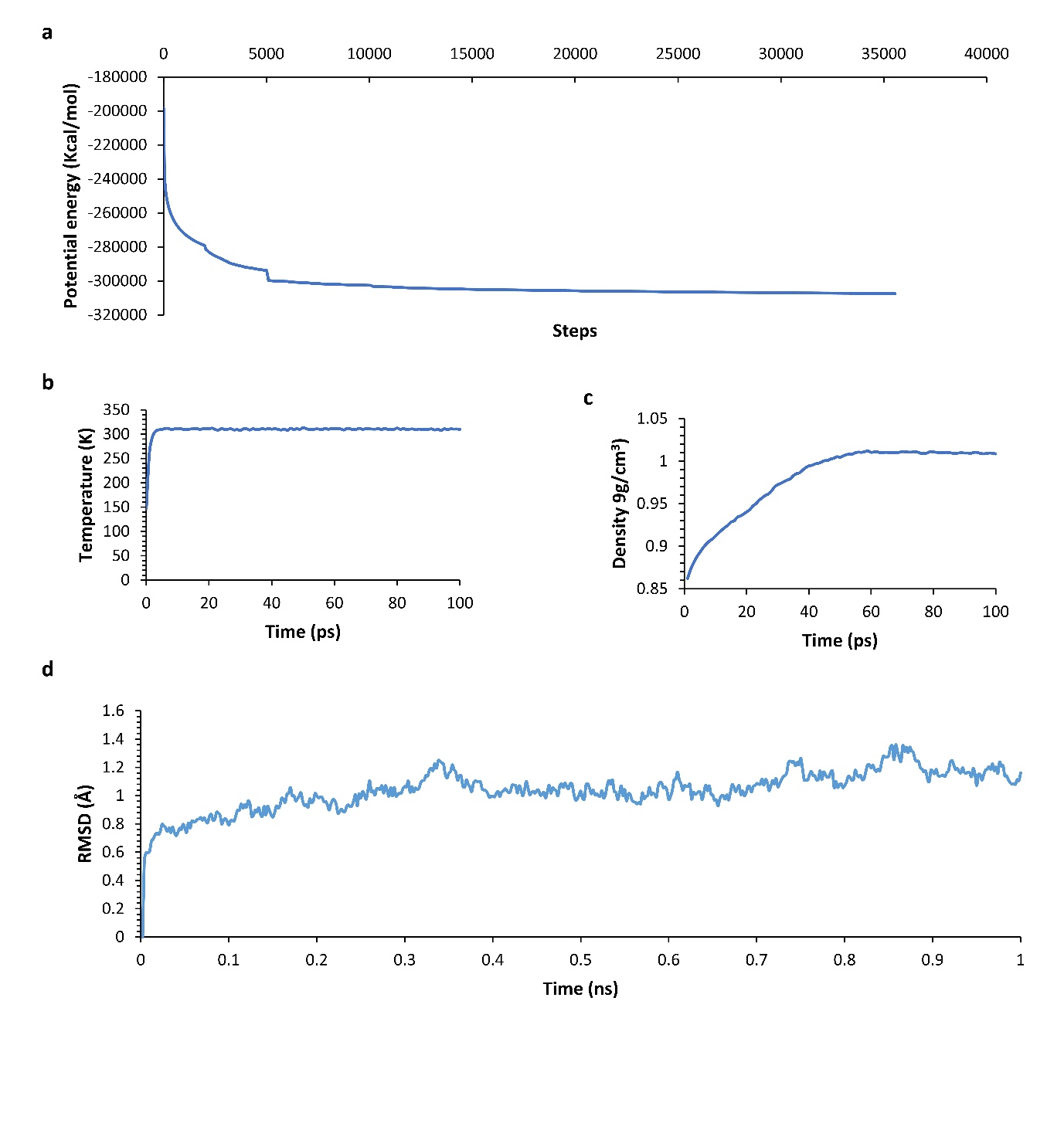


**Figure S4**: (a) Potential energy vs. Time, (b) Temperature vs. Time, (c) Density vs. Time and (d) RMSD of protein backbone of BChE-DNP complex.


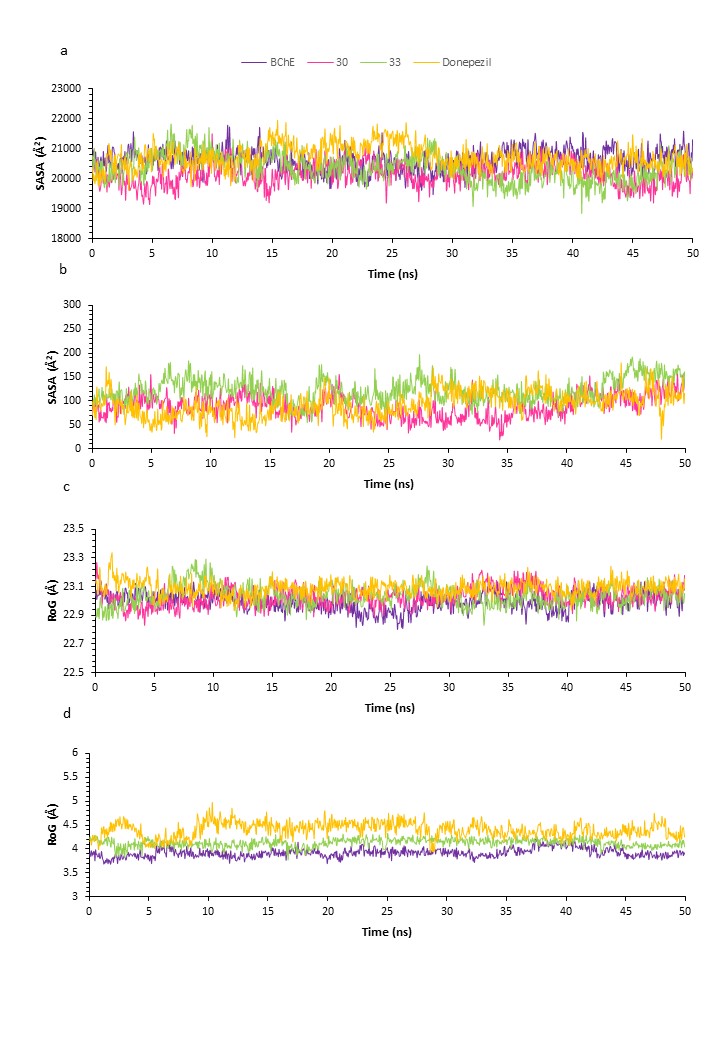


**Figure S5**: (a) SASA of protein-ligand complexes, (b) SASA of the ligands, (c) RoG of protein-ligand complexes and (d) RoG of the ligands.


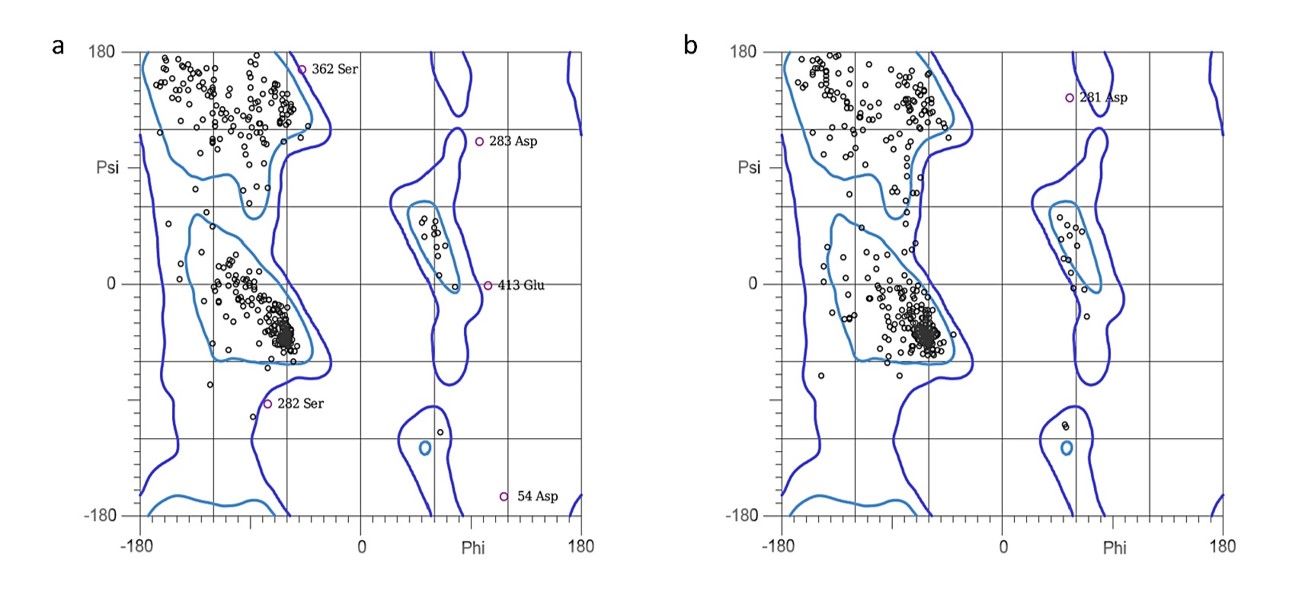


**Figure S6**: Ramachandran plot of the rat BChE.
